# Short communication: Antibacterial effects of essential oils from *Cinnamomum cassia* bark and *Eucalyptus globulus* leaves–The involvements of major constituents

**DOI:** 10.1371/journal.pone.0288787

**Published:** 2023-07-14

**Authors:** Ha Thi Thanh Nguyen, Atsushi Miyamoto, Hai Thanh Nguyen, Huong Thi Pham, Hong Thi Hoang, Ngoc Thi My Tong, Linh Thi Ngoc Truong, Ha Thi Thu Nguyen

**Affiliations:** 1 Department of Veterinary Pharmacology, Faculty of Veterinary Medicine, Vietnam National University of Agriculture, Hanoi, Vietnam; 2 Department of Veterinary Pharmacology, Joint Faculty of Veterinary Medicine Kagoshima University, Kagoshima, Japan; 3 Department of Plant Biotechnology, Faculty of Biotechnology, Vietnam National University of Agriculture, Hanoi, Vietnam; Universidad Autonoma de Chihuahua, MEXICO

## Abstract

Essential oils from *Cinnamomum cassia* bark and *Eucalyptus globulus* leaves have been traditionally applied for bacterial infections, through both of aromatherapy and oral application. (E)-cinnamaldehyde and 1,8 cineole have been identified as their major secondary metabolites, and are also generally considered as the main active ingredients responsible for their medicinal applications. However, ethnobotanical doctors still prefer to use whole essentials oils over purified compounds in bacterial infections. We therefore hypothesized that multi-compound extracts might exert better effects than isolated ingredients. In order to verify the hypothesis about advantages of whole materials, we examined antibacterial properties of the 2 plant essential oils in the comparison with their isolated major compounds, such as (E)-cinnamaldehyde and 1,8 cineole. Effects of liquid- and vapor-phase were examined on a set of 6 gram-positive and -negative bacteria, applying broth dilution, agar well diffusion and disc volatilization methods. In all 3 investigations, we observed that whole cinnamon and eucalyptus oils, with the lower concentrations of (E)*-*cinnamaldehyde (89.1%) and 1,8 cineole (61.2%), were able to induce better effects than the purified active compounds (≥ 99%). These results partly explain the advantages of using whole essential oils over isolated ingredients, and therefore support the application of traditional dosage forms for bacterial infections in ethnomedicine.

## Introduction

*Cinnamomum cassia* bark (CC) and *Eucalyptus globulus* leaves (EG) essential oils have been traditionally used to treat bacterial infections, through both of aromatherapy and oral application [1]. Even though their active components are available, ethnobotanical doctors still prefer to use whole essential oils [1]. Synergisms between different constituents in one plant have been proposed for several therapeutic functions, such as in cases of *Glycyrrhiza uralensis* or *Gingko biloba*, when researchers observed that crude extracts could exert stronger effects than the maximum effects of isolated compounds, in platelet aggregation [2] or anti-hepatitis C virus activities [3]. Similarly, one of our previous studies that investigated the effects of *Artemisia vulgaris* L. on basilar arteries and identified serotonin as an active component responsible for tissue contraction, also demonstrated that total effects of the plant were not solely mediated by this ingredient, because antagonisms on all tissue serotonin receptors could not eliminate the extracts’ effects [4]. The concepts that whole plant materials are more advantageous than isolated active ingredients also underpin the philosophy of herbal medicine [2]. However, there have been no studies investigated effects of CC and EG essential oils in the comparison with their major ingredients on a set of bacteria to generally propose such kind of hypothesis for antibacterial properties. (E)-cinnamaldehyde is the major compound of cinnamon essential oil, occupying from 68.5 to 90.08% [5, 6], and 1,8 cineole is the major compound of eucalyptus essential oil, occupying from 49.07 to 83.59% [7]. We therefore examined antibacterial effects of the 2 plant oils, along with purified (E)-cinnamaldehyde and 1,8 cineole, so as to verify the hypothesis about advantages of whole plant materials over isolated compounds.

## Materials and methods

### Plant essential oil and compounds

CC and EG essential oils were supplied by Vuon Duoc Lieu Herbarium, Vietnam National University of Agriculture. Plant identities were confirmed by Dr Tho Thi Bui, based on the voucher specimens that have been deposited at Vietnam National University of Agriculture. Essential oils were obtained through hydro-distillation, and the provider certified that products reached the Vietnam National Standards for herbal essential oils used as medicine, established in Vietnamese Pharmacopoeia by the Ministry of Health, which require the content of (E)-cinnamaldehyde in cinnamon bark oil is not less than 85% [8], and that of 1,8 cineole in eucalyptus leave oil is not less than 60% [9]. All compounds and reagents at analytical standards were purchased from Sigma-Aldrich (St, Louis, MO, USA). In order to observe dose-dependent effects, dimethyl sulfoxide (DMSO) was applied to dilute essential oils and purified compound (start from 100%) to obtain serial tested concentrations, calculated as percentage volume (% v/v)

### Gas chromatography analysis of (E)-cinnamaldehyde and 1,8 cineole

Analysis of (E)-cinnamaldehyde and 1,8 cineole in CC and EG essential oil were performed by GC-FID techniques, followed the General Method of using Gas chromatography on capillary columns for the analysis of essential oils, established by Vietnam Standards and Quality Institute (code TCVN 9653:2013, issued date: 10^th^, April, 2013) [10]. Briefly, gas chromatography of hexane diluted standards (E-cinnamaldehyde; 1,8 cineole) or essential oils were performed on a GC-1310 gas chromatograph (Thermo Fisher Scientific Inc., USA), equipped with a Agilent GC Column DB-5 (30m × 0.25mm × 0.25μm), and connected to a flame ionization detector (FID). The column temperature was 120°C. The injector port and detector temperature were 250°C (split ratio: 1/10). Nitrogen (99.999% purity) constant pressure 120 kPa was employed as carrier gas. The analyzed sample volume was 1 μl. The oven temperature was programmed at 80°C for 5 min, then 20°C/ min to 260°C, and then left at 260°C for 5 min. The contents of (E)-cinnamaldehyde in CC and 1,8 cineole in EG essential oils were determined by comparing the areas of the peaks in sample profiles with those of the (E)-cinnamaldehyde and 1,8 cineole standards. Analysis was performed in triplicate.

### Bacterial strains

Tested bacteria, including 2 gram-positive (gram (+)), such as *Bacillus subtilis* ATCC 6633 (*B*. *sub*), *Staphylococcus aureus* ATCC 25923 (*S*. *aureus*), and 4 gram-negative (gram (-)), such as *Escherichia coli* (*E*. *coli*) ATCC 25922, *E*. *coli* ATCC 85922, *Pseudomonas aeruginosa* ATCC 9027, *Salmonella typhimurium* ATCC 13311, were purchased from the American Type Culture Collection(ATCC, Rockville, MD, USA).

### Effects of vapor-phase on bacteria

Effects of vapor-phase on bacteria were evaluated by the disc volatilization assay (also known as the inverted petri plate method), followed the methods described by Houdkova et al. [11] and Lopez et al. [12], with some modifications. A glass petri dish (90 mm diameter, 20 mm height) containing 25 ml Muller Hinton agar inoculated with bacteria at the concentration of 10^5^ cfu/ml was used, and each 100 μL of tested materials at established concentration (100%, 50% or 25%) was impregnated to 10 mm sterile filter discs placed in the center of medium-free cover. Because the thickness that 25 ml agar produced in 90 mm diameter petri dish was 8 mm, the free atmosphere above the growing microorganism was calculated as 76 cm^3^, and the concentrations of 100 μL tested materials at 100%, 50% and 25% were converted to 1316 μL/ L; 658 μL/ L and 329 μL/ L, respectively. These plates were then immediately inverted on top of the lead and sealed with sterile adhesive tape to prevent any leakage of vapors to the atmosphere. The petri dishes were incubated under 37°C for 24 h. Generally, antimicrobials agents diffuse from the disc to the atmosphere inside the petri dishes and then to the agar, which inhibits the growth of tested bacteria [11]. The diameters of inhibitory zones, which had no visible bacterial growth were regarded as a measure of their antimicrobial activity [11]. Experiments were performed in triplicate.

### Effects of liquid-phase on bacteria

The effects of liquid-phase were evaluated through broth dilution and agar well diffusion methods.

Broth dilution method was performed to determine minimum inhibitory concentration (MIC) values of plant extracts, followed the methods established by Clinical and Laboratory Standards Institute [13] and Mogana et al. [14], with some modification. Tested solutions were mixed with Muller Hinton broth (MHB) in 96-well microplate to produce serial dilutions ranged from 25.0% to 0.39%. Final bacterial concentration was adjusted as 5x10^5^ cfu/ml. Ninety-six well plate sealing films were then applied to prevent the evaporation of oils or oil compounds. All bacteria were incubated at 37°C for 24 h. The lowest concentration displaying no visible growth was recorded as the MIC. DMSO served as a negative control and kanamycin was applied as a positive and quality control, with MIC against *E*. *coli* ATCC 25922 was determined as 2 μg/ml, which was within the acceptable limits (from 1–4 μg/ml) established by Clinical and Laboratory Standards Institute [13].

Agar well diffusion method was performed followed Gonelimali et al. [15], and with some modification. Briefly, Muller Hinton agar plate was inoculated with bacteria at the final concentration of 10^6^ cfu/ml, and a hole with a diameter of 10 mm was punched aseptically with a cork borer. One hundred μL of tested materials, including essential oils and compounds at established concentrations, was added into the well. Twelve mm diameter caps were then applied to cover the 10 mm wells to prevent any evaporation of oils or oil compounds. Agar plates were incubated under 37°C for 24 h and inhibitory zones (excluding 10 mm of well diameter) were measured. Kanamycin revealed a clear concentration-dependent inhibitory zones and was applied as a positive control, while DMSO induced no inhibition and was applied as a negative control. Experiments were performed in triplicate.

### Statistical analysis

Results are expressed as means ± standard deviation (S.D). Statistical analyses were performed by unpaired *t* test or the Bonferroni test after one-way analysis of variance (one-way ANOVA). Significance was established when the probability level was equal to or less than 5%.

## Results

### Gas chromatography analysis of (E)-cinnamaldehyde and 1,8 cineole

**Fig 1:**
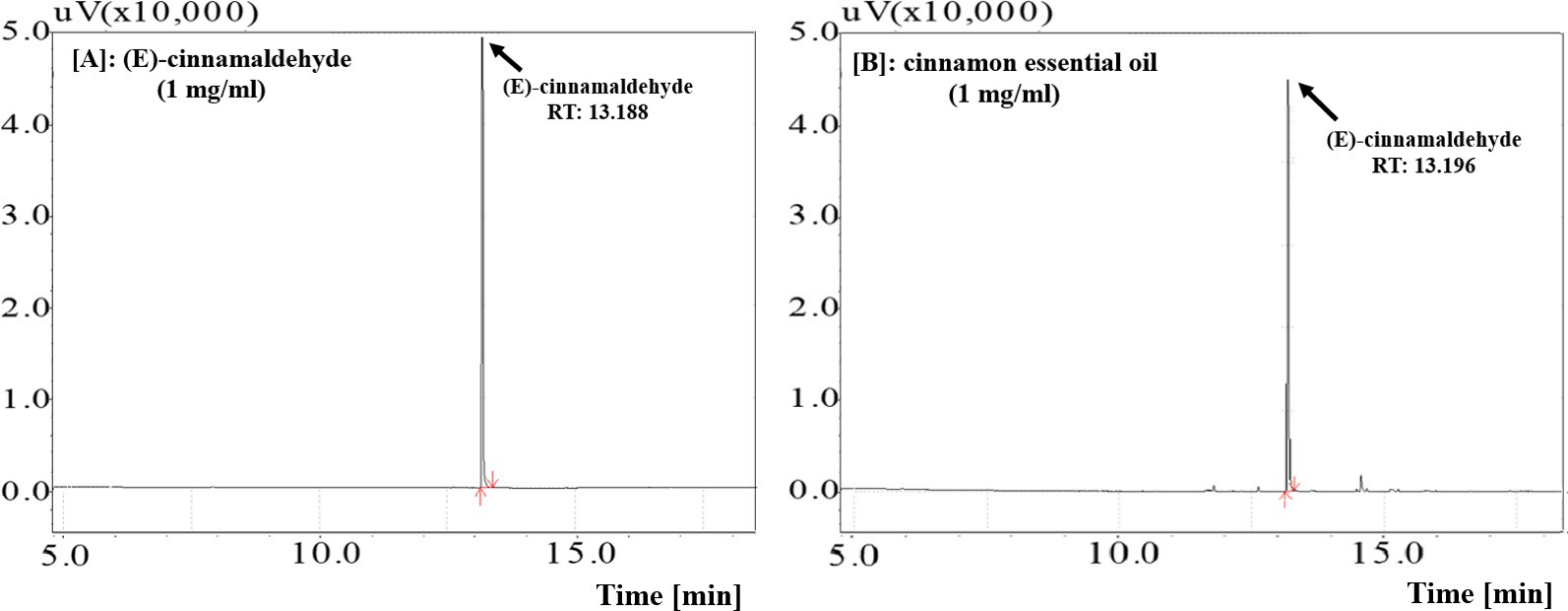
Representative GC-FID chromatogram of [A] (E)-cinnamaldehyde standard (1 mg/ml) and [B] cinnamon essential oil (1 mg/ml).

The representative gas chromatography profiles of (E)-cinnamaldehyde and CC essential oil are shown in Fig 1A and 1B. The retention time (RT) of the peak in CC essential oil (13.196 min, Fig 1B) was similar to that of the (E)-cinnamaldehyde (13.188 min, Fig 1A). By compared the area of this peak with that of the standard, (E)-cinnamaldehyde content of CC was determined as 89.1 ± 0.31%.

**Fig 2:**
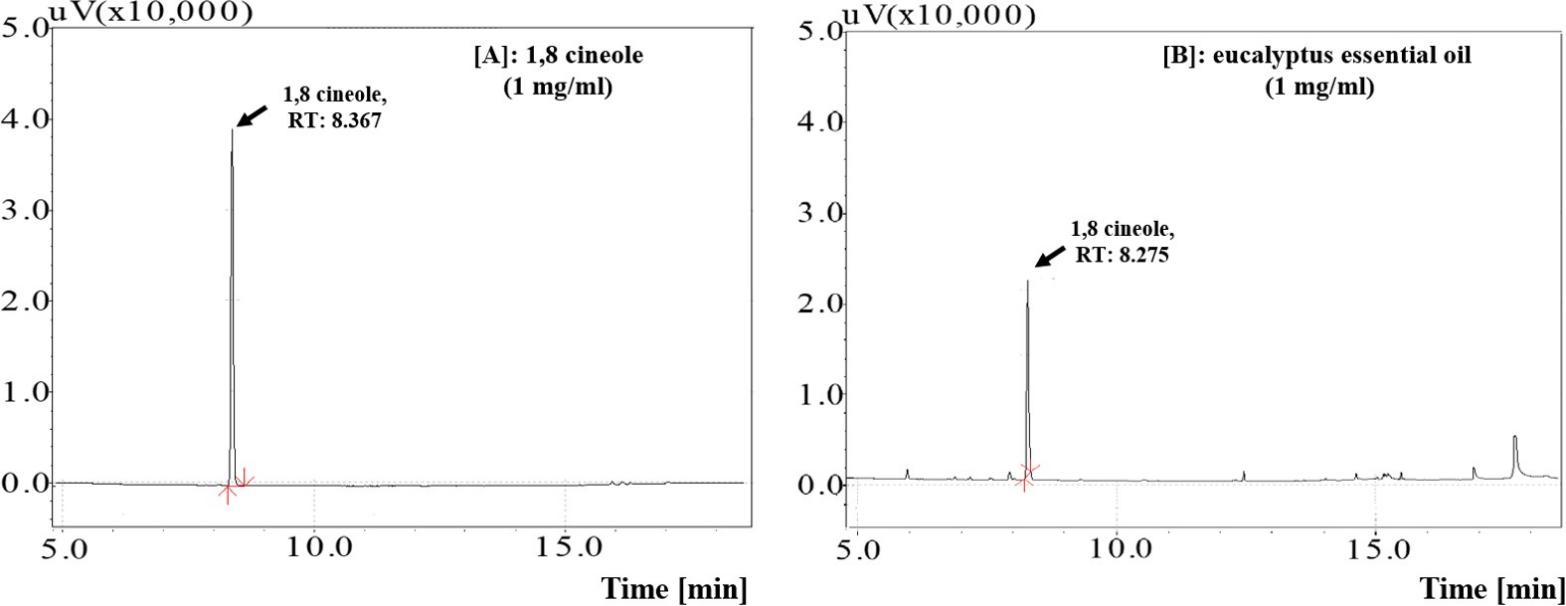
Representative GC-FID chromatogram of [A] 1,8 cineole standard (1 mg/ml) and [B] eucalyptus essential oil (1 mg/ml).

The representative gas chromatography profiles of 1,8 cineole and EG essential oil are shown in Fig 2A and 2B. The RT of peak in EG essential oil (8.275 min, Fig 2B) was similar to that of the 1,8 cineole (8.367 min, Fig 2A). By compared the area of this peak with that of the standard, 1,8 cineole content of EG was determined as 61.20 ± 0.15%.

### Antibacterial effects of vapor-phase

**Fig 3:**
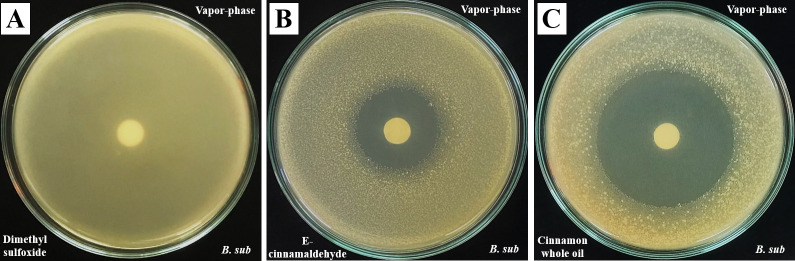
Inhibitory zones induced by vapor-phase of 100 μL [A] Dimethyl sulfoxide, [B] (E)-cinnamaldehyde 100% (equal to 1316 μL/ L) and [C] *Cinnamomum cassia* whole oil 100% (equal to 1316 μL/ L) on *Bacillus subtilis* ATCC 6633. *B*. *sub*, *Bacillus subtilis* ATCC 6633.

Antibacterial effects of vapor-phase, as expressed by inhibitory zones induced with the disc volatilization method, are shown in Table 1 and Fig 3.

**Table 1:** Inhibition zones (mm) induced by vapor-phase of plant essential oils and isolated compounds on bacteria.

Tested material	Conc (μL/L)	Gram (+) bacterium		Gram (-) bacterium			
		*B*. *sub*	*S*. *aureus*	*E*. *coli* 25922	*E*. *coli* 85922	*P*. *aeruginosa*	*S*. *typhimurium*
*Cinnamomum cassia* oil	1316	**42.4** ^**a**^ ^*^ **±0.9**	**33.5** ^**a**^ ^*^ **±0.6**	**15.1** ^**a**^ ^*^ **±0.9**	**12.8** ^**a**^ ^*^ **±1.0**	-	-
	658	35.8^b^±0.3	32.1^b^ ± 1.4	8.4^b^ ± 0.4	6.4^b^ ± 0.9	-	-
	329	25.8^c^ ± 0.3	15.3^c^ ± 1.1	-	-	-	-
(E)-cinnamaldehyde	1316	**27.8**^**a**^ **± 1.1**	**23.3**^**a**^ **± 0.7**	**7.9**^**a**^ **± 1.5**	-	-	-
	658	12.4^b^ ± 1.0	11.5^b^ ± 0.8	4.4^b^ ± 0.6	-	-	-
	329	8.7^c^ ± 0.7	5.1^c^ ± 0.6	-	-	-	-

Results are expressed as means ± standard deviation (S.D) of 3 tests.—means no inhibition. Values with different superscript letters indicate significant differences (P<0.05) compared with other values from different concentrations of the same materials, assessed with the Bonferroni test after one-way analysis of variance (one-way ANOVA). Values with superscripted

* and bold letters indicate significant difference (p <0.05) between the maximum inhibition zone induced by essential oil vs. that of the respective plant compound (*Cinnamomum cassia* oil vs. (E)-cinnamaldehyde), assessed by unpaired *t* test. *B*. *sub*, *Bacillus subtilis* ATCC 6633; *E*. *coli*, *Escherichia coli; S*. *aureus*, *Staphylococcus aureus* ATCC 25923; *S*. *typhimurium*, *Salmonella typhimurium* ATCC 13311; *P*. *aeruginosa*, *Pseudomonas aeruginosa* ATCC 9027.

In vapor-phase, CC and (E)-cinnamaldehyde exerted a clear dose dependent inhibition on both gram bacteria, while EG and 1,8 cineole induced no effects. In addition, we observed that at same concentrations, inhibitory zones induced by (E)-cinnamaldehyde were significantly smaller than those induced by CC crude essential oil on *B*. *sub* (27.8 ± 1.1 vs. 42.4 ± 0.9 mm and Fig 3B vs 3C), *S*. *aureus* (23.3 ± 0.7 vs. 33.5 ± 0.6 mm), and two *E*. *coli* strains (7.9 ± 1.5 vs. 15.1 ± 0.9 mm for ATCC 25922 and no inhibition vs. 12.8 ± 1.0 mm for ATCC 85922).

### Antibacterial effects of liquid-phase

Antibacterial effects of liquid-phase, as expressed by MIC values, are shown in Table 2.

**Table 2:** Minimum inhibition concentration (percentage volume: % v/v) of plant essential oils and isolated compounds on bacteria.

Tested material	Conc (dilute ratio)	Gram (+) bacterium		Gram (-) bacterium			
		*B*. *sub*	*S*. *aureus*	*E*. *coli* 25922	*E*. *coli* 85922	*P*. *aeruginosa*	*S*. *typhimurium*
*Cinnamomum cassia* oil		1.56	1.56	3.13	3.13	12.5	12.5
(E)-cinnamaldehyde		1.56	1.56	6.25	6.25	12.5	12.5
*Eucalyptus globulus* oil		3.13	3.13	-	-	-	-
1,8 cineole		6.25	6.25	-	-	-	-

- means no inhibition. *B*. *sub*, *Bacillus subtilis* ATCC 6633; *E*. *coli*, *Escherichia coli; S*. *aureus*, *Staphylococcus aureus* ATCC 25923; *S*. *typhimurium*, *Salmonella typhimurium* ATCC 13311; *P*. *aeruginosa*, *Pseudomonas aeruginosa* ATCC 9027.

**Fig 4:**
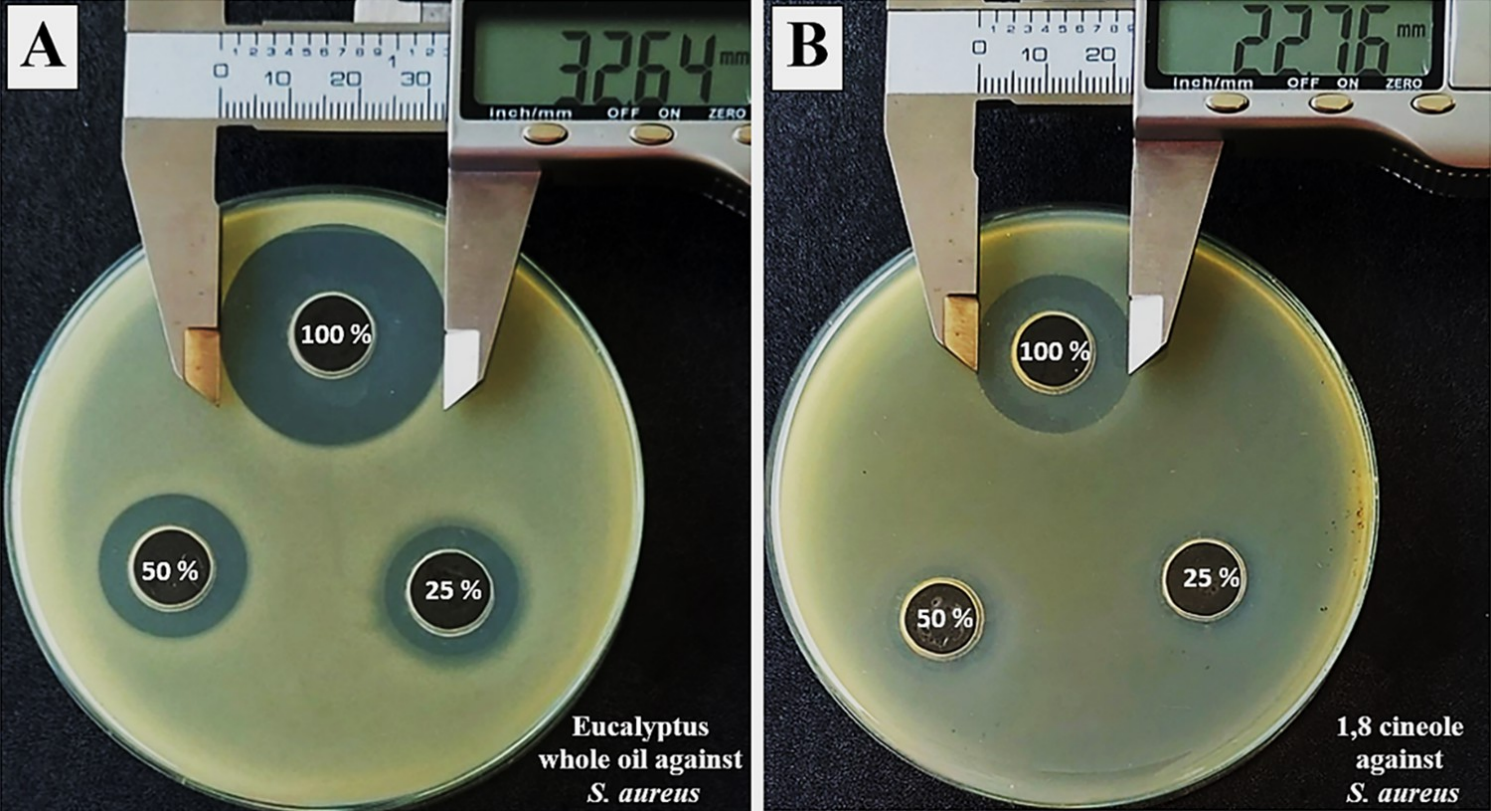
Inhibitory zones induced by liquid-phase of [A] *Eucalyptus globulus* whole essential oil and [B] 1,8 cineole on *Staphylococcus aureus* ATCC 25923. *S*. *aureus*, *Staphylococcus aureus* ATCC 25923.

From Table 2, we observed that liquid-phase of CC was superior to EG, exerting effects on both gram bacteria, while EG showed effects on only gram (+) ones. Crude extracts of CC and EG had stronger effects than the major isolated compounds, as evidenced by lower MIC values. Specifically, MICs of CC against two *E*. *coli* strains were 3.13%, lower than those of (E)-cinnamaldehyde (6.25%), and MICs of EG against *B*. *sub* and *S*. *aureus* were 3.13%, lower than those of 1,8 cineole (6.25%). Antibacterial effects of liquid-phase were further confirmed with agar well diffusion methods, and results are shown in Table 3 and Fig 4.

**Table 3:** Inhibition zones (mm) induced by liquid-phase of plant essential oils and isolated compounds on bacteria.

Tested material	Conc (%)	Gram (+) bacterium		Gram (-) bacterium			
		*B*. *sub*	*S*. *aureus*	*E*. *coli* 25922	*E*. *coli* 85922	*P*. *aeruginosa*	*S*. *typhimurium*
*Cinnamomum cassia* oil	100	**20.7** ^**a**^ ^*^ **±0.8**	**25.7** ^**a**^ ^*^ **± 1.2**	**20.7** ^**a**^ ^*^ **±0.2**	**20.0** ^**a**^ ^*^ **±1.2**	14.3^a^ ±0.3	11.3 ^a^ ±0.8
	50	13.4^b^ ± 0.6	14.9^b^ ± 0.4	13.2^b^ ± 1.0	10.2^b^ ± 1.1	10.7^b^ ± 1.3	7.3^b^ ± 0.5
	25	5.7^c^ ± 0.3	5.8^c^ ± 0.3	-	-	-	-
(E)-cinnamaldehyde	100	**16.3**^**a**^ **± 0.5**	**21.8**^**a**^ **± 0.9**	**16.7**^**a**^ **±1.3**	**16.1**^**a**^ **±0.6**	14.8^a^ ±0.6	11.3^a^ ± 0.1
	50	10.6^b^ ± 1.0	12.8^b^ ± 0.6	11.1 ^b^ ± 0.2	8.6 ^b^ ± 1.2	8.8 ^b^ ± 0.2	6.8 ^b^ ± 0.8
	25	3.5^c^ ± 0.6	3.4^c^ ± 0.4	-	-	-	-
*Eucalyptus globulus* oil	100	**16.4** ^**a**^ ^*^ **± 1.5**	**22.4** ^**a**^ ^*^ **± 0.4**	-	-	-	-
	50	7.1^b^ ± 0.2	8.9^b^ ± 0.7	-	-	-	-
	25	4.0^c^ ± 0.7	5.8^c^ ± 0.4	-	-	-	-
1,8 cineole	100	**8.5 ± 0.6**	**12.1**^**a**^ **± 0.6**	-	-	-	-
	50	-	4.3^b^ ± 0.7	-	-	-	-

Results are expressed as means ± standard deviation (S.D) of 3 tests.—means no inhibition. Values with different superscript letters indicate significant differences (P<0.05) compared with other values from different concentrations of the same materials, assessed with the Bonferroni test after one-way analysis of variance (one-way ANOVA). Values with superscripted

* and bold letters indicate significant difference (p <0.05) between the maximum inhibition zone induced by essential oil vs. that of the respective plant compound (*Cinnamomum cassia* oil vs. (E)-cinnamaldehyde, *Eucalyptus globulus* oil vs. 1,8 cineole), assessed by unpaired *t* test. *B*. *sub*, *Bacillus subtilis* ATCC 6633; *E*. *coli*, *Escherichia coli; S*. *aureus*, *Staphylococcus aureus* ATCC 25923; *S*. *typhimurium*, *Salmonella typhimurium* ATCC 13311; *P*. *aeruginosa*, *Pseudomonas aeruginosa* ATCC 9027.

Similar to results obtained with broth dilution methods, agar well diffusion methods also showed that CC exerted effects on both gram bacteria, while EG had effects on only gram (+) ones. At same concentrations, inhibitory zones induced by liquid-phase of CC were significantly larger than those induced by (E)-cinnamaldehyde on *B*. *sub* (20.7 ± 0.8 vs. 16.3 ± 0.5 mm); *S*. *aureus* (25.7 ± 1.2 vs. 21.8 ± 0.9 mm) and two *E*. *coli* strains (20.7 ± 0.2 vs. 16.7 ± 1.3 mm for ATCC 25922 and 20.0 ± 1.2 vs. 16.1 ± 0.6 mm for ATCC 85922). However, these differences were not observed in cases of *P*. *aeruginosa* and *S*. *typhimurium*. Liquid-phase of EG and 1,8 cineole exerted effects on only gram (+) bacteria, in which EG were superior, as evidenced by the significantly larger inhibitory zones on both *B*. *sub* (16.4 ± 1.5 vs. 8.5 ± 0.6 mm) and *S*. *aureus* (22.4 ± 0.4 vs. 12.1 ± 0.6 mm and Fig 4A vs. 4B).

## Discussion

Antibacterial effects observed with vapor- and liquid-phase of CC and EG essential oil partly explain their traditional uses for bacterial infections, through both of aromatherapy and oral application. Our study showed that effects of CC were superior than EG, as the former exerted effects on both gram bacteria, while the later had effects on only gram (+) ones, those are usually more sensitive due to the structure of cell walls. These results were similar to previous studies, which reported that CC had broad-spectrum antibacterial activities [5], while EG were hardly active against gram (-) bacteria [16]. The broad-spectrum antibacterial nature of CC oil might be partly explained by its ability to increase membrane permeability, and therefore attacks both gram bacteria in similar manners [5]. Interestingly, CC crude extracts induced stronger effects than those obtained with (E)-cinnamaldehyde, in both of vapor- and liquid-phase, suggesting that antibacterial effects of CC might be partly, but not solely, attributed to the (E)-cinnamaldehyde constituents. Similarly, liquid-phase of EG oil exerted inhibition that stronger than those of purified 1,8 cineole, its major ingredient. (E)-cinnamaldehyde and 1,8 cineole are considered as required chemotypes of CC and EG essential oils, and Ministry of Health applies their contents as criteria to decide whether or not a crude essential oil from plant materials reaches the requirements to be used as herbal medicine [8, 9]. However, Lis-Balchin et al. [17] observed that there were no significant correlations between 1,8 cineole contents and antibacterial activities of commercial essential oils from eucalyptus trees, and EG oil with 91% of 1,8 cineole was less antibacterial than *Eucalyptus radiata* oil with 84% of 1,8 cineole, suggesting that the higher concentrations of active compounds were not always represented the higher effects on all therapeutic functions. In fact, one herbal plant might have various biological activities and therefore could be applied in different remedies, based on the diversity of its constituents. However, it is likely that major compounds play different roles in each pharmacological function [1]. For examples, eugenol, a major compound in *Syzygium aromaticum* and clove essential oil, respectively occupying 52.53% and 66.81% of the total extracts, was found to exert similar anti-fungal and antitrypanosomal activities with the crude oil [18, 19], but was stronger in antioxidant activities, while, in contrast, was less potent against bacteria [19]. These observations suggested that it is necessary to verify roles of active compounds in accordance with the target pharmacological activities. In cases of CC and EG, there have been several studies examined the effects of isolated major ingredients in comparison with whole essential oils on therapeutic functions different from antibacterial properties, and researchers also reported various comparative results, depending on investigated effects. For examples, essential eucalyptus oil was less effective in anxiolytic effects than isolated 1,8 cineole ingredient, and researchers therefore proposed the purified compound as a better anxiety relief therapy [20]. In contrast, the whole oil was observed to exert stronger antioxidant, anti-inflammatory, anti-proliferative and antiviral activities than 1,8 cineole by other studies [21, 22]. Similarly, purified cinnamaldehyde, while exerted equal effects with cinnamon oils in anti-cancer activities [23], was observed to be less effective in several anti-inflammatory parameters [24], but showed stronger activities in the anti-tyrosinase, anti-melanogenic and xanthine oxidase inhibitory effects [25, 26]. Our study is the first one that aimed to examine the effects of CC and EG essential oils along with purified major ingredients on a set of bacteria to generally compare their antibacterial properties. Superior effects observed with whole extracts over the isolated compounds in both vapor- and liquid-phase suggest the important involvement of minor ingredients in the plants’ total effects. Our results, together with previous researches about other pharmacological functions of CC and EG essential oils [20–26], confirm that while higher contents of major active constituents, including (E)-cinnamaldehyde and 1,8 cineole, are generally considered to represent higher therapeutic qualities of plant materials [1, 8, 9], they might not be able to exert equal effects with the whole extracts if applied as isolated drugs. On the other hand, our study also observed that while CC oil were more potent than (E)*-*cinnamaldehyde against *B*. *sub*, *S*. *aureus* and 2 *E*. *coli* strains, it only exerted similar activities against *P*. *aeruginosa* and *S*. *typhimurium*, suggesting that the comparison results were also dependent on bacteria. Similar to our study, previous researchers have reported that antibacterial properties of plant compounds were highly strain-specific selective, as they produced largely different effects, even against bacteria of the same genera [27]. Based on our results, we suggest that whole essential oils from the 2 tested plant materials might be better than their isolated major compounds as a treatment for bacterial infections, but further studies are necessary to confirm the *in vivo* effects and standardize their apply in traditional medicine.

## Conclusion

In conclusion, superior effects of CC and EG essential oils over the isolated major compounds observed with this study partly give scientific evidences explaining the advantages of using whole plant materials in several bacterial infections.

## Limitations

Our study has several limitations are noted for future work. Although higher *in vitro* effects of whole extracts to the main compounds have been confirmed, mechanisms responsible for this mode of actions have not yet been identified. Secondly, *in vivo* studies are still necessary to examine these advantages in the treatment for patients with bacterial infections.

## Supporting information

S1 Data (RAR)

## Abbreviations

CC: *Cinnamomum cassia* bark
EG: *Eucalyptus globulus* leaves
DMSO: dimethyl sulfoxide
Gram-positive: gram (+)
Gram-negative: gram (-)
MIC: minimum inhibitory concentration
B. sub: *Bacillus subtilis* ATCC 6633
E. coli: *Escherichia coli*
S. aureus: *Staphylococcus aureus* ATCC 25923
